# Associations between ERα/β gene polymorphisms and osteoporosis susceptibility and bone mineral density in postmenopausal women: a systematic review and meta-analysis

**DOI:** 10.1186/s12902-018-0230-x

**Published:** 2018-02-20

**Authors:** Heping Zhu, Jiannong Jiang, Qiang Wang, Jun Zong, Liang Zhang, Tieliang Ma, Youjia Xu, Leiyan Zhang

**Affiliations:** 1 Department of Orthopedics, The Affiliated Yixing Hospital of Jiangsu University, Yixing, 214200 China; 2 Department of Orthopedics, The Second Affiliated Hospital of Soochow University, Suzhou, 215004 China; 3 Department of Orthopedics, Northern Jiangsu People’s Hospital, Yangzhou, 225001 China; 4 Central Laboratory, The Affiliated Yixing Hospital of Jiangsu University, Yixing, 214200 China

**Keywords:** Estrogen receptor, Postmenopausal osteoporosis, Gene polymorphism, Meta-analysis

## Abstract

**Background:**

Many studies have reported associations between estrogen receptor (ER) gene polymorphisms and postmenopausal osteoporosis (PMOP) risk and bone mineral density (BMD), but the results are controversial. The aim of the present meta-analysis is to verify the association between ERα and ERβ gene polymorphisms and osteoporosis susceptibility and BMD in postmenopausal women.

**Methods:**

PubMed, EMBASE, Web of Science, the Cochrane Library and China WeiPu Library were searched. OR and WMD with 95% CI were calculated to assess the association.

**Results:**

Overall, no significant association was observed between ERα *Xba*I, ERα *Pvu*II and PMOP susceptibility in either overall, Caucasian or Asian populations. ERα G2014A was significantly associated with a decreased risk of PMOP in Caucasian populations. There was a significant association between ERβ *Rsa*I and PMOP risk in both overall and Asian populations. Caucasian PMOP women with ERα *Xba*I XX and Xx genotypes had a higher LS Z value than women with xx genotype. ERα *Xba*I XX genotype was associated with increased FN BMD in overall and Caucasian populations, an increased FN Z value in Asians, and a decreased FN Z value in Caucasians. There was also a significant association between ERα *Xba*I Xx genotype and an increased FN Z value in either Asians or Caucasians. ERα *Pvu*II PP genotype was associated with a low LS Z value in Caucasians and a low FN BMD and Z value in Asians. Pp genotype in PMOP women was significantly correlated with low LS BMD in overall populations, a low FN Z value in either overall, Caucasian or Asian populations.

**Conclusion:**

Each ERα and ERβ gene polymorphism might have different impact on PMOP risk and BMD in various ethnicities.

## Background

Postmenopausal osteoporosis (PMOP) is a common metabolic bone disorder characterized by low bone mineral density (BMD) and increased fracture risks [1–3]. It is estimated that osteoporosis affects approximately 10 million American adults, with another 34 million being at high risk due to low bone mass [4].

The pathophysiology of PMOP is considered as a disorder or negative imbalance of bone metabolism and remodeling, with bone resorption outpacing bone formation [3], suggesting that vitamin D and parathyroid hormone (PTH) and other factors related to bone resorption and formation may play a key role in the underlying mechanism and pathophysiology of PMOP [5–8]. Furthermore, genetic factors including genes and gene polymorphisms may also play an important role in the development of PMOP [9].

Estrogen is another important hormone that plays an important role in the pathogenesis of PMOP, knowing that reduced ovarian production of estrogen after menopause is a cause for the initial phase of rapid bone loss and osteoporosis in women [3]. Estrogen is known as an important regulator of bone metabolism, and estrogen deficiency is believed to be the cause of BMD loss, increased mechanical loading-induced bone remodeling, and the development of PMOP [10]. Knowing that the action of estrogen is predominantly mediated by estrogen receptor (ER), including ERα and ERβ by binding to different ligands to mediate various biological effects [3, 10], more attention has been paid to the relationship between ERs and PMOP risk and BMD in postmenopausal women [11–38]. However, the results of studies currently available about this issue are controversial.

Previous meta-analyses have been performed to assess the pooled effects of ER gene polymorphisms on BMD and fracture risk [39–41]. WANG et al. [39] showed that the ERα *Xba*I (rs9340799) polymorphism was associated with BMD at diverse skeletal sites, and ERα *Pvu*II (rs2234693) PP genotype played a role in protecting the lumbar spine but on the other hand might be a risk factor for the femoral neck fracture. However, to the best of our knowledge, no meta-analysis has been performed to explore the relationships between ER gene [ERα *Xba*I (rs9340799), ERα *Pvu*II (rs2234693) and ERα G2014A (rs2228480)] and ERβ gene [ERβ *Alu*I (rs4986938) and ERβ *Rsa*I (rs1256049)] polymorphisms and PMOP susceptibility and BMD of the lumbar spine and femoral neck in postmenopausal women. To address these issues, we performed a meta-analysis of all currently available studies relating ER gene [ERα *Xba*I (rs9340799), ERα *Pvu*II (rs2234693) and ERα G2014A (rs2228480)] and ERβ gene [ERβ *Alu*I (rs4986938) and ERβ *Rsa*I (rs1256049)] polymorphisms with PMOP risk and BMD.

## Methods

### Data sources and searches

We searched PubMed, EMBASE, Web of Science, the Cochrane Library and China WeiPu Library to identify case-control studies that investigated the associations between ERα gene polymorphisms [ERα *Xba*I (rs9340799), ERα *Pvu*II (rs2234693) and ERα G2014A (rs2228480)] ERβ gene polymorphisms [ERβ *Alu*I (rs4986938) and ERβ *Rsa*I (rs1256049)] and osteoporosis susceptibility and BMD in postmenopausal women by using the following search terms (‘PMOP’ OR ‘Postmenopausal osteoporosis’ OR ‘Postmenopausal’) AND (‘Estrogen Receptor’ OR ‘ER’) AND (‘polymorphism’ OR ‘single nucleotide polymorphism’ OR ‘SNP’ OR ‘variation’). To analyze the pooled effects of ER gene polymorphisms on BMD, the following search terms were used: (‘PMOP’ OR ‘Postmenopausal osteoporosis’ OR ‘Postmenopausal’) AND (‘Estrogen Receptor’ OR ‘ER’) AND (‘polymorphism’ OR ‘single nucleotide polymorphism’ OR ‘SNP’ OR ‘variation’) AND (‘BMD’ OR ‘bone mineral density’). Then, one-by-one screening was performed by two authors according to the inclusion and exclusion criteria. No language restrictions were applied. Secondary searches of eligible studies were conducted by searching the reference lists of the selected studies, reviews or comments.

### Inclusion and exclusion criteria

The inclusion criteria of our meta-analysis are as follows: (1) case-control studies; (2) studies on BMD and fracture risks in postmenopausal women with PMOP due to estrogen deficiency using postmenopausal women without PMOP or healthy volunteers as control; (3) studies reporting alleles and genotypes of at least one of the ER gene polymorphisms in women with or without PMOP: ERα *Xba*I (rs9340799), ERα *Pvu*II (rs2234693), ERα G2014A (rs2228480), ERβ *Alu*I (rs4986938) and ERβ *Rsa*I (rs1256049); (3) studies reporting the sample size, mean and standard deviation (SD) of BMD (g/cm^2^) or BMD Z value in PMOP women with at least one of the ER genotypes; and (4) studies with sufficient data. The exclusion criteria were: (1) reviews or case reports without controls, and (2) studies with no availability of current data; and (3) duplicated reports.

### Data extraction

Data from the eligible studies were extracted according to the inclusion and exclusion criteria by two authors, and a consensus was reached by discussion. In the study of associations between ER gene polymorphisms and PMOP risk, the following data were collected: author list, year of publication, ethnicity, sample size, alleles, genotype of each gene polymorphism and Hardy-Weinberg equilibrium (HWE). The following data were collected for analysis of differences in BMD in PMOP women with various ER genotypes: author list, year of publication, ethnicity, the number of cases and mean and SD of BMD (g/cm^2^) and BMD Z value.

### Data synthesis and statistical analysis

We calculated odds ratios (OR) and 95% confidence interval (CI) to evaluate the association between ER gene polymorphisms and PMOP risk (osteoporosis occurred in postmenopausal women due to estrogen deficiency as represented by low BMD and increased fracture risks). The strength of association between ER gene polymorphisms and PMOP susceptibility was evaluated by OR and 95% CI under the allele contrast model, heterozygote model, homozygote model, dominant model and recessive model. HWE was calculated in the control population to evaluate the quality of the data by using chisquare test. Regarding the associations between BMD and ER gene polymorphisms, we compared BMD (g/cm^2^) and BMD Z value in PMOP women under the heterozygote and homozygote model respectively using the weight mean difference (WMD) and 95% CI. Heterogeneity of the included studies was examined by a chi-squared-based Q statistical test and quantified by I2 metric value. If I2 value was > 50% or *P* < 0.10, ORs and WMD were pooled by the random effect model; otherwise, the fixed effect model was used. Power analysis was performed using the Power and Precision V4 software (Biostat Inc., Englewood, USA). Sensitivity analysis was performed to assess the impact of each study on the combined effect of the present meta-analysis. Besides, subgroup analysis was also performed according to the ethnicity of the study populations. Stata 12.0 software (StataCorp, College Station, TX, USA) was used and a *P <* 0.05 was considered as statistically significant.

## Results

### Study selection and characteristics

**Fig. 1:**
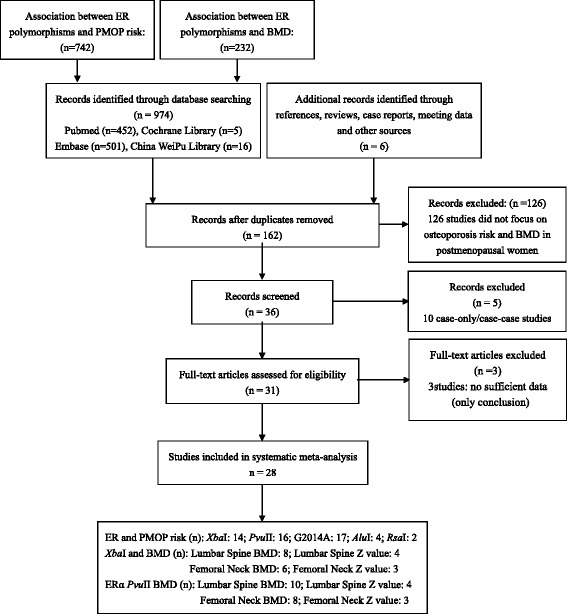
Flow chart showing the process of selection

A total of 28 studies [11–38] were finally recruited in our meta-analysis. The study selection and inclusion process is shown in Fig. 1. Fourteen studies [11–24] reported the association between ERα *Xba*I and PMOP risk, and the number of the included studies that reported the alleles and genotypes of ERα *Pvu*II, ERα G2014A, ERβ *Alu*I and ERβ *Rsa*I was 16 [11–25, 32], 4 [26–29], 4 [17, 30–32] and 2 [30, 31], respectively. Ivanova et al. [20], Albagha et al. [33], Aerssens et al. [24], Kurt et al. [34], Ge et al. [36] and Pérez et al. [19] reported both the lumbar spine and femoral neck BMD (g/cm^2^). Jeedigunta et al. [15] and Kurabayashi et al. [35] were also recruited in the assessment of the lumbar spine BMD (g/cm^2^) in ERα *Xba*I genotypes. Ivanova et al. [20], Albagha et al. [33] and An et al. [38] reported both the lumbar spine and femoral neck Z values. Shang et al. [11] also studied the lumbar spine Z value in PMOP with ERα *Xba*I genotypes. Ten studies [15, 19, 20, 23, 24, 33–37] and 8 studies [19, 20, 23, 24, 33, 34, 36, 37] were recruited in the pooled analysis of differences in lumbar spine and femoral neck BMD (g/cm^2^) in PMOP women carrying ERα *Pvu*II, respectively. With regard to differences in lumbar spine and femoral neck Z value in PMOP women with ERα *Pvu*II, 4 studies [11, 20, 33, 38] and 3 studies [20, 33, 38] were included in our meta-analysis, respectively. In addition, all these studies complied with HWE. The characteristics of the included studies are shown in Tables 1, 2 and 3.

**Table 1:** General characteristics of studies assciated with postmenopausal osteoporosis risk

Author	Year	Ethnicity	Sample Size		ERα *Xba*I										HWE
					Case					Control					
			Case	Control	X	x	XX	Xx	xx	X	x	XX	Xx	xx	
Shang et al.	2016	Asian	198	276	338	58	146	46	6	109	443	10	89	177	0.77
Wang et al.	2015	Asian	72	72	125	19	55	15	2	132	12	62	8	2	0.21
Li et al.	2014	Asian	440	791	254	626	31	192	217	404	1178	48	308	435	0.50
Erdogan et al.	2011	Caucasian	50	30	41	59	7	27	16	28	32	6	16	8	0.70
Jeedigunta et al.	2010	Asian	247	254	253	241	60	133	54	306	202	81	144	29	0.32
Tanriover et al.	2010	Caucasian	50	50	48	52	5	38	7	54	46	12	30	8	0.14
Harsløf et al.	2010	Caucasian	228	225	134	322	19	96	113	164	286	30	104	91	0.97
Musumeci et al.	2009	Caucasian	100	200	130	70	35	60	5	155	245	13	129	58	0.26
Pérez et al.	2008	Caucasian	64	68	48	80	9	30	25	46	90	5	36	27	0.13
Ivanova et al.	2007	Caucasian	220	180	256	184	73	110	37	163	197	25	113	42	0.58
Huang et al.	2006	Asian	66	116	19	113	2	15	49	46	186	4	38	74	0.74
Nam et al.	2005	Asian	6	168	0	12	0	0	6	63	273	6	51	111	0.96
Qin et al.	2004	Asian	244	273	120	368	11	98	135	137	409	13	111	149	0.18
Aerssens et al.	2000	Caucasian	135	239	92	178	14	64	57	175	303	32	111	96	0.99
Author	Year	Ethnicity	Sample Size		ERα *Pvu*II										HWE
					Case					Control					
			Case	Control	P	p	PP	Pp	pp	P	p	PP	Pp	pp	
Shang et al.	2016	Asian	198	276	156	240	28	100	70	386	166	138	110	28	0.38
Wang et al.	2015	Asian	60	60	30	90	3	24	33	32	88	3	26	31	0.40
Li et al.	2014	Asian	440	791	368	512	65	238	137	498	1084	69	360	362	0.12
Sonoda et al.	2012	Asian	114	171	118	110	24	70	20	137	205	31	75	65	0.26
Erdogan et al.	2011	Caucasian	50	30	42	58	8	26	16	38	22	10	18	2	0.11
Jeedigunta et al.	2010	Asian	247	254	181	313	50	81	116	232	276	60	112	82	0.08
Tanriover et al.	2010	Caucasian	50	50	39	61	7	25	18	48	52	14	20	16	0.79
Harsløf et al.	2010	Caucasian	228	224	198	258	46	106	76	233	215	63	107	54	0.52
Musumeci et al.	2009	Caucasian	100	200	120	80	30	60	10	186	214	31	124	45	0.53
Pérez et al.	2008	Caucasian	64	68	56	72	11	34	19	58	78	12	34	22	0.86
Ivanova et al.	2007	Caucasian	220	180	226	214	58	110	52	148	212	21	106	53	0.37
Morón et al.	2006	Caucasian	87	175	79	95	17	45	25	171	179	45	81	49	0.33
Huang et al.	2006	Asian	66	116	79	53	23	33	10	68	164	11	46	59	0.64
Nam et al.	2005	Asian	6	168	2	10	1	0	5	130	206	25	80	63	0.96
Qin et al.	2004	Asian	244	273	193	295	40	113	91	223	323	43	137	93	0.52
Aerssens et al.	2000	Caucasian	135	239	120	150	27	66	42	219	259	47	125	67	0.41
Author	Year	Ethnicity	Sample Size		ERα G2014A										HWE
					Case					Control					
			Case	Control	A	G	AA	GA	GG	A	G	AA	GA	GG	
Wajanavisit et al.	2015	Asian	99	113	94	104	33	28	38	179	47	72	35	6	0.53
Gómez et al.	2007	Caucasian	70	500	30	110	2	26	42	303	697	40	223	237	0.21
Ongphiphadhanakul et al.	2003	Asian	33	325	23	43	5	13	15	129	521	13	103	209	0.94
Ongphiphadhanakul et al.	2001	Asian	106	122	56	156	8	40	58	37	207	2	33	87	0.57
Author	Year	Ethnicity	Sample Size		ERβ *Alu*I										HWE
					Case					Control					
			Case	Control	A	G	AA	GA	GG	A	G	AA	GA	GG	
Shoukry et al.	2015	Caucasian	200	180	223	177	75	73	52	125	235	30	65	85	0.46
Huang et al.	2015	Asian	413	890	678	148	285	108	20	1384	396	541	302	47	0.57
Harsløf et al.	2010	Caucasian	228	224	154	302	26	102	100	186	262	35	116	73	0.32
Morón et al.	2006	Caucasian	88	177	76	100	11	54	23	146	208	34	78	65	0.23
Author	Year	Ethnicity	Sample Size		ERβ *Rsa*I										HWE
					Case					Control					
			Case	Control	A	G	AA	GA	GG	A	G	AA	GA	GG	
Shoukry et al.	2015	Caucasian	200	180	52	348	2	48	150	37	323	1	35	144	0.47
Huang et al.	2015	Asian	413	777	329	497	63	203	147	759	795	169	421	187	0.28

**Table 2:** Characteristics of included studies of lumbar spine BMD, femoral neck BMD, lumbar spine Z value and femoral neck Z value in ERα *Xba*I genotypes

ERα *Xba*I			Lumbar Spine BMD (g/cm^2^)						ERα *Xba*I			Femoral Neck BMD (g/cm^2^)					
			XX		Xx		xx					XX		Xx		xx	
Author	Year	Ethnicity	N	Mean ± SD	N	Mean ± SD	N	Mean ± SD	Author	Year	Ethnicity	N	Mean ± SD	N	Mean ± SD	N	Mean ± SD
Ivanova et al.	2007	Caucasian	73	0.75 ± 0.17	110	0.81 ± 0.06	37	0.87 ± 0.07	Ivanova et al.	2007	Caucasian	73	0.69 ± 0.08	110	0.69 ± 0.04	37	0.65 ± 0.03
Albagha et al.	2001	Caucasian	27	0.88 ± 0.03	89	0.88 ± 0.02	90	0.85 ± 0.02	Albagha et al.	2001	Caucasian	27	0.77 ± 0.03	89	0.73 ± 0.01	90	0.72 ± 0.02
Aerssens et al.	2000	Caucasian	14	0.94 ± 0.21	64	0.93 ± 0.22	57	0.88 ± 0.16	Aerssens et al.	2000	Caucasian	14	0.73 ± 0.03	64	0.68 ± 0.09	57	0.70 ± 0.20
Jeedigunta et al.	2010	Asian	60	0.89 ± 0.15	133	0.86 ± 0.13	54	0.64 ± 0.16	Kurt et al.	2012	Caucasian	41	0.79 ± 0.09	94	0.8 ± 0.08	40	0.83 ± 0.10
Kurt et al.	2012	Caucasian	41	0.95 ± 0.12	94	0.92 ± 0.12	40	0.93 ± 0.10	Ge et al.	2006	Asian	37	0.70 ± 0.10	134	0.68 ± 0.07	26	0.67 ± 0.07
Kurabayashi et al.	1999	Asian	1	1.18 ± 0.00	20	0.92 ± 0.04	61	0.92 ± 0.02	Pérez et al.	2008	Caucasian	7	0.59 ± 0.02	36	0.58 ± 0.01	20	0.56 ± 0.02
Ge et al.	2006	Asian	37	0.73 ± 0.08	134	0.74 ± 0.09	26	0.75 ± 0.13									
Pérez et al.	2008	Caucasian	7	0.70 ± 0.02	31	0.67 ± 0.02	24	0.66 ± 0.02									
ERα *Xba*I			Lumbar Spine Z value						ERα *Xba*I			Femoral Neck Z value					
			XX		Xx		xx					XX		Xx		xx	
Author	Year	Ethnicity	N	Mean ± SD	N	Mean ± SD	N	Mean ± SD	Author	Year	Ethnicity	N	Mean ± SD	N	Mean ± SD	N	Mean ± SD
Shang et al.	2016	Asian	146	−1.98 ± 0.91	146	−1.65 ± 0.02	6	−0.35 ± 2.19	Ivanova et al.	2007	Caucasian	73	−2.00 ± 0.00	110	−2.00 ± 0.00	37	−1.90 ± 0.00
Ivanova et al.	2007	Caucasian	73	−2.10 ± 0.00	110	−0.6 ± 0.00	37	−0.1 ± 0.00	Albagha et al.	2001	Caucasian	27	−2.00 ± 0.23	89	−0.42 ± 0.10	90	−0.52 ± 0.12
Albagha et al.	2001	Caucasian	27	−0.34 ± 0.20	89	−0.29 ± 0.11	90	−0.47 ± 0.11	An et al.	2000	Asian	10	0.42 ± 0.57	84	0.11 ± 0.66	152	−0.32 ± 0.76
An et al.	2000	Asian	10	0.48 ± 0.49	84	0.12 ± 0.85	152	−0.26 ± 0.58

**Table 3:** Characteristics of included studies of lumbar spine BMD, femoral neck BMD, lumbar spine Z value and femoral neck Z value in ERα *Pvu*II genotypes

ERα *Pvu*II			Lumbar Spine BMD (g/cm^2^)						ERα *Pvu*II			Femoral Neck BMD (g/cm^2^)					
			PP		Pp		pp					PP		Pp		pp	
Author	Year	Ethnicity	N	Mean ± SD	N	Mean ± SD	N	Mean ± SD	Author	Year	Ethnicity	N	Mean ± SD	N	Mean ± SD	N	Mean ± SD
Ivanova et al.	2007	Caucasian	58	0.70 ± 0.09	110	0.71 ± 0.10	52	0.77 ± 0.06	Ivanova et al.	2007	Caucasian	58	0.52 ± 0.02	110	0.68 ± 0.01	52	0.76 ± 0.05
Albagha et al.	2001	Caucasian	37	0.87 ± 0.03	102	0.86 ± 0.02	67	0.88 ± 0.02	Albagha et al.	2001	Caucasian	37	0.75 ± 0.02	102	0.71 ± 0.01	67	0.75 ± 0.02
Aerssens et al.	2000	Caucasian	27	0.93 ± 0.18	66	0.91 ± 0.22	42	0.89 ± 0.17	Aerssens et al.	2000	Caucasian	27	0.69 ± 0.06	66	0.70 ± 0.09	42	0.69 ± 0.11
Jeedigunta et al.	2010	Asian	50	0.92 ± 0.18	81	0.89 ± 0.11	116	0.81 ± 0.14	Kurt et al.	2012	Caucasian	44	0.77 ± 0.08	104	0.81 ± 0.09	46	0.82 ± 0.09
Kurt et al.	2012	Caucasian	44	0.93 ± 0.13	104	0.93 ± 0.11	46	0.93 ± 0.09	Ge et al.	2006	Asian	38	0.68 ± 0.09	93	0.67 ± 0.07	67	0.69 ± 0.08
Kurabayashi et al.	1999	Asian	19	0.99 ± 0.04	27	0.89 ± 0.03	36	0.91 ± 0.02	Ge et al.	2006	Asian	38	0.68 ± 0.09	92	0.67 ± 0.08	67	0.69 ± 0.08
Ge et al.	2006	Asian	38	0.73 ± 0.10	93	0.74 ± 0.09	67	0.75 ± 0.10	Qin et al.	2004	Asian	40	0.57 ± 0.01	113	0.60 ± 0.01	91	0.59 ± 0.01
Ge et al.	2006	Asian	38	0.73 ± 0.10	92	0.74 ± 0.09	67	0.75 ± 0.10	Pérez et al.	2008	Caucasian	9	0.59 ± 0.01	37	0.57 ± 0.01	16	0.57 ± 0.02
Qin et al.	2004	Asian	40	0.70 ± 0.01	113	0.70 ± 0.01	91	0.72 ± 0.01									
Pérez et al.	2008	Caucasian	11	0.73 ± 0.03	34	0.66 ± 0.02	17	0.65 ± 0.02									
ERα *Pvu*II			Lumbar Spine Z value						ERα *Pvu*II			Femoral Neck Z value					
			PP		Pp		pp					PP		Pp		pp	
Author	Year	Ethnicity	N	Mean ± SD	N	Mean ± SD	N	Mean ± SD	Author	Year	Ethnicity	N	Mean ± SD	N	Mean ± SD	N	Mean ± SD
Shang et al.	2016	Asian	28	−1.54 ± 0.35	100	−1.67 ± 0.91	70	−2.79 ± 1.46	Ivanova et al.	2007	Caucasian	58	−2.00 ± 0.00	110	−1.90 ± 0.00	52	−0.70 ± 0.00
Ivanova et al.	2007	Caucasian	58	−2.40 ± 0.00	110	−2.10 ± 0.00	52	−1.50 ± 0.00	Albagha et al.	2001	Caucasian	37	−0.29 ± 0.17	102	−0.59 ± 0.09	67	−0.28 ± 0.15
Albagha et al.	2001	Caucasian	37	−0.35 ± 0.16	102	−0.44 ± 0.10	67	−0.28 ± 0.14	An et al.	2000	Asian	53	−0.48 ± 0.90	128	−0.19 ± 0.80	128	0.31 ± 0.49
An et al.	2000	Asian	53	−0.53 ± 0.16	128	−0.21 ± 0.99	65	0.22 ± 0.46									

### Power analysis

Before initiation of the meta-analysis, a power analysis was conducted by using the Power and Precision V4 software to verify whether the included studies could offer adequate power (> 80%). The result showed that the statistical power in our study was sufficient to detect the associations between ER gene polymorphisms and PMOP risk.

### Associations between ER gene polymorphisms and PMOP risk

**Fig. 2:**
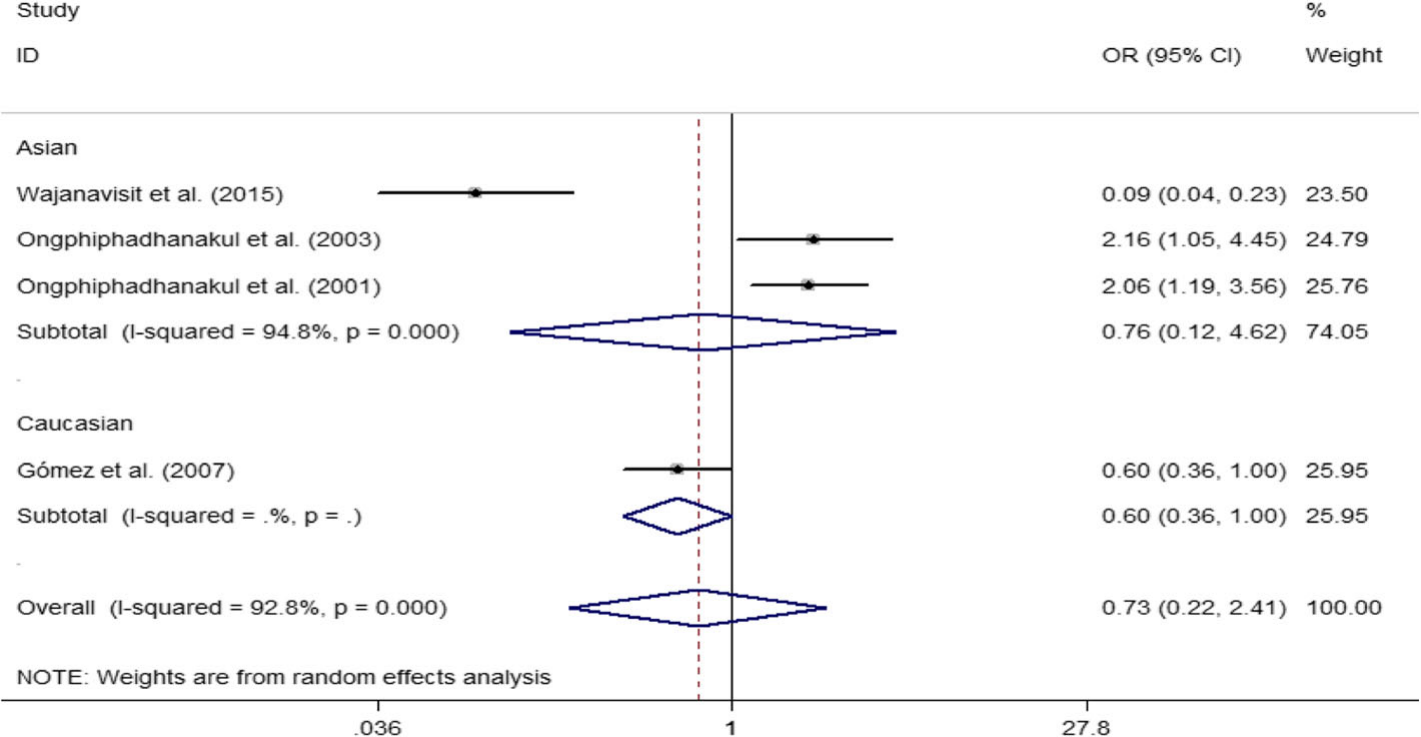
Forest plot describing the meta-analysis under the dominant model for the association between ERα G2014A polymorphism and the risk of PMOP (GA/AA vs. GG)

Overall, we did not find any significant association between ERα *Xba*I and ERα *Pvu*II polymorphisms and risk of PMOP in either overall, Caucasian or Asian populations (all *P* > 0.05) (Table 4). ERα G2014A polymorphism played a protcetive role in developing PMOP in Caucasian populations, while no significant association was observed in overall and Asian populations (both *P* > 0.05). All the data are shown in Table 4 and Fig. 2.

**Table 4:** Results of genetic models for ERα *Xba*I, ERα *Pvu*II, ERα G2014A, ERβ *Alu*I and ERβ *Rsa*I polymorphisms and osteoporosis susceptibility in postmenopausal women

Comparison	N	Test of association			Model	Test of heterogeneity		Begg’s test	Egger’s test
		OR	95% CI	*P* value		*P* value	I^2^ (%)	*P* value	*P* value
ERα *Xba*I									
Overall	14								
X vs. x		1.21	0.73–2.00	0.455	R	< 0.001	96.4	0.584	0.955
XX vs. xx		1.84	0.71–4.75	0.206	R	< 0.001	93.7	0.443	0.465
Xx vs. xx		1.19	0.83–1.70	0.357	R	< 0.001	80.1	0.511	0.610
Xx/XX vs. xx		1.34	0.82–2.18	0.240	R	< 0.001	90.4	0.661	0.545
XX vs. Xx/xx		1.50	0.70–3.24	0.296	R	< 0.001	93.4	0.443	0.875
Caucasian	7								
X vs. x		1.15	0.76–1.74	0.510	R	< 0.001	88.0		
XX vs. xx		1.56	0.56–4.39	0.399	R	< 0.001	88.9		
Xx vs. xx		1.13	0.76–1.67	0.540	R	0.021	59.8		
Xx/XX vs. xx		1.24	0.76–2.01	0.387	R	< 0.001	76.2		
XX vs. Xx/xx		1.30	0.56–3.03	0.536	R	< 0.001	88.2		
Asian	7								
X vs. x		1.23	0.47–3.25	0.668	R	< 0.001	98.0		
XX vs. xx		2.18	0.37–12.73	0.388	R	< 0.001	98.1		
Xx vs. xx		1.22	0.63–2.36	0.553	R	< 0.001	88.0		
Xx/XX vs. xx		1.39	0.56–3.46	0.481	R	< 0.001	94.6		
XX vs. Xx/xx		1.77	0.44–7.14	0.424	R	< 0.001	96.0		
ERα *Pvu*II									
Overall	16								
P vs. p		0.96	0.71–1.29	0.769	R	< 0.001	92.3	0.753	0.616
PP vs. pp		0.99	0.55–1.78	0.961	R	< 0.001	90.8	1.000	0.886
Pp vs. pp		1.01	0.72–1.41	0.956	R	< 0.001	82.3	0.753	0.501
PP/Pp vs. pp		0.97	0.65–1.43	0.868	R	< 0.001	88.7	0.893	0.539
PP vs. Pp/pp		0.99	0.65–1.53	0.977	R	< 0.001	87.3	0.893	0.976
Caucasian	8								
P vs. p		0.95	0.71–1.26	0.716	R	< 0.001	79.2		
PP vs. pp		0.93	0.49–1.79	0.831	R	< 0.001	81.4		
Pp vs. pp		0.98	0.73–1.31	0.877	R	0.112	40.0		
PP/Pp vs. pp		0.97	0.67–1.39	0.861	R	0.008	63.5		
PP vs. Pp/pp		0.97	0.59–1.58	0.895	R	< 0.001	78.2		
Asian	8								
P vs. p		0.97	0.57–1.66	0.919	R	< 0.001	95.6		
PP vs. pp		1.08	0.40–2.96	0.877	R	< 0.001	94.4		
Pp vs. pp		1.04	0.58–1.88	0.889	R	< 0.001	90.2		
PP/Pp vs. pp		0.98	0.50–1.95	0.962	R	< 0.001	93.8		
PP vs. Pp/pp		1.05	0.50–2.20	0.891	R	< 0.001	91.8		
ERα G2014A									
Overall	4								
A vs. G		0.89	0.32–2.51	0.825	R	< 0.001	95.1	0.308	0.237
AA vs. GG		0.88	0.08–9.19	0.912	R	< 0.001	92.9	0.734	0.419
GA vs. GG		0.76	0.28–2.03	0.581	R	< 0.001	88.1	0.734	0.530
GA/AA vs. GG		0.73	0.22–2.41	0.601	R	< 0.001	92.8	0.734	0.530
AA vs. GA/GG		1.13	0.23–5.72	0.878	R	< 0.001	88.6	0.734	0.299
Caucasian	1								
A vs. G		0.63	0.41–0.96	0.032	R	–	–		
AA vs. GG		0.28	0.07–1.21	0.089	R	–	–		
GA vs. GG		0.66	0.39–1.11	0.116	R	–	–		
GA/AA vs. GG		0.60	0.36–1.00	0.050	R	–	–		
AA vs. GA/GG		0.34	0.08–1.43	0.141	R	–	–		
Asian	3								
A vs. G		1.00	0.23–4.46	0.996	R	< 0.001	96.6		
AA vs. GG		1.28	0.05–30.10	0.878	R	< 0.001	95.2		
GA vs. GG		0.77	0.17–3.45	0.736	R	< 0.001	91.3		
GA/AA vs. GG		0.76	0.12–4.62	0.765	R	< 0.001	94.8		
AA vs. GA/GG		1.69	0.20–14.27	0.630	R	< 0.001	92.2		
ERβ *Alu*I									
Overall	4								
A vs. G		1.25	0.78–2.00	0.362	R	< 0.001	91.5	1.000	0.997
AA vs. GG		1.27	0.52–3.13	0.597	R	< 0.001	88.4	0.734	0.647
GA vs. GG		1.16	0.65–2.07	0.606	R	0.001	81.0	0.734	0.408
GA/AA vs. GG		1.29	0.66–2.53	0.459	R	< 0.001	87.8	0.734	0.612
AA vs. GA/GG		1.21	0.65–2.24	0.553	R	< 0.001	85.7	0.497	0.646
Caucasian	3								
A vs. G		1.23	0.58–2.57	0.590	R	< 0.001	94.3		
AA vs. GG		1.28	0.34–4.84	0.717	R	< 0.001	92.2		
GA vs. GG		1.30	0.60–2.78	0.504	R	0.001	86.5		
GA/AA vs. GG		1.36	0.55–3.39	0.507	R	< 0.001	91.8		
AA vs. GA/GG		1.10	0.37–3.22	0.863	R	< 0.001	90.3		
Asian	1								
A vs. G		1.31	1.06–1.62	0.012	R	–	–		
AA vs. GG		1.24	0.72–2.13	0.441	R	–	–		
GA vs. GG		0.84	0.48–1.48	0.548	R	–	–		
GA/AA vs. GG		1.10	0.64–1.87	0.739	R	–	–		
AA vs. GA/GG		1.44	1.12–1.84	0.004	R	–	–		
ERβ *Rsa*I									
Overall	2								
A vs. G		0.92	0.50–1.70	0.785	R	0.010	85.0		
AA vs. GG		0.49	0.34–0.70	< 0.001	F	0.261	20.9		
GA vs. GG		0.87	0.41–1.84	0.722	R	< 0.001	85.9		
GA/AA vs. GG		0.85	0.37–1.95	0.704	R	< 0.001	88.9		
AA vs. GA/GG		0.66	0.48–0.90	0.009	F	0.408	0		
Caucasian	1								
A vs. G		1.30	0.83–2.04	0.245	R	–	–		
AA vs. GG		1.92	0.17–21.41	0.596	F	–	–		
GA vs. GG		1.32	0.80–2.15	0.273	R	–	–		
GA/AA vs. GG		1.33	0.82–2.17	0.246	R	–	–		
AA vs. GA/GG		1.81	0.16–20.11	0.630	F	–	–		
Asian	1								
A vs. G		0.69	0.58–0.82	< 0.001	R	–	–		
AA vs. GG		0.47	0.33–0.68	< 0.001	F	–	–		
GA vs. GG		0.61	0.47–0.81	< 0.001	R	–	–		
GA/AA vs. GG		0.57	0.44–0.74	< 0.001	R	–	–		
AA vs. GA/GG		0.65	0.47–0.89	0.007	F	–	–		

*R* Random effect model

*F* Fixed effect model

**Fig. 3:**
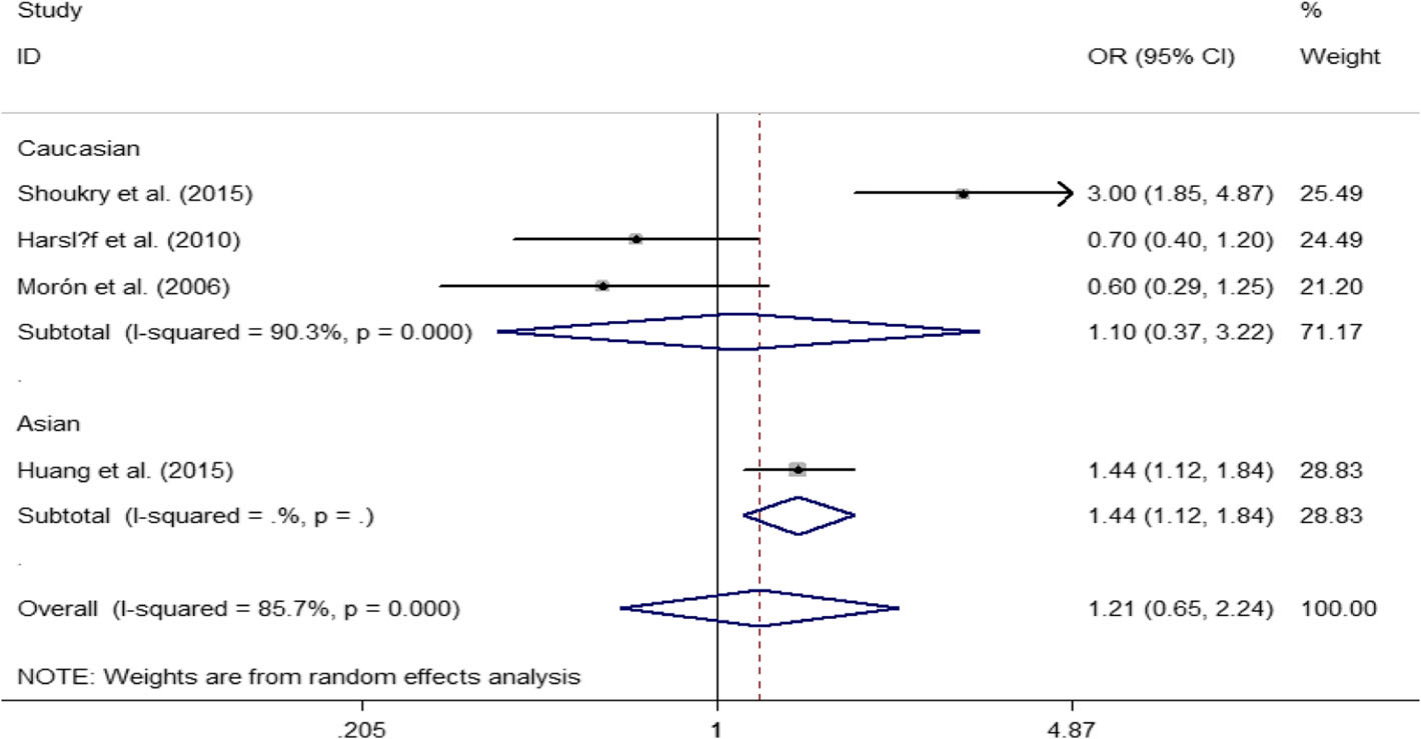
Forest plot describing the meta-analysis under the recessive model for the association between ERβ *Alu*I polymorphism and the risk of PMOP (AA vs. GA/GG)

With regard to ERβ polymorphism, ERβ *Alu*I was significantly associated with the risk of developing PMOP in Asian postmenopausal women under the recessive model; however, we did not observe any significant association between ERβ *Alu*I and PMOP risk in overall and Caucasian populations (both *P* > 0.05) (Table 4 and Fig. 3). Furthermore, we also found that there was a remarkable association between ERβ *Rsa*I polymorphism and decreased PMOP risk in overall and Asian populations (Table 4).

### Associations between ER gene polymorphisms and BMD in PMOP women

#### ERα *Xba*I and lumbar spine bone mineral density (BMD g/cm^2^ and BMD Z value)

In our meta-analysis, no significant difference in lumbar spine BMD (g/cm^2^) was observed between PMOP women with ERα *Xba*I XX, ERα *Xba*I Xx and ERα *Xba*I xx genotype in either overall, Caucasian or Asian populations (all *P* > 0.05) (Table 5). The lumbar spine BMD Z value in Caucasian PMOP women carrying ERα *Xba*I XX genotype was greater than that in those carrying xx genotype, while no significant difference was observed in overall and Asian populations (both *P* > 0.05). ERα *Xba*I Xx genotype was found to be significantly associated with high lumbar spine BMD Z value in either overall or Caucasian populations but not in Asian populations.

**Table 5:** Meta-analysis of differences of Lumbar Spine BMD, Femoral Neck BMD, Lumbar Spine Z value and Femoral Neck Z value between each genotype of ERα *Xba*I and ERα *Pvu*II polymorphism

ERα *Xba*I	XX vs. xx						Xx vs. xx					
	Test of differences			Model	Test of heterogeneity		Test of differences			Model	Test of heterogeneity	
	N	WMD (95% CI)	*P* value		*P* value	I^2^ (%)	N	WMD (95% CI)	*P* value		*P* value	I^2^ (%)
Lumbar Spine BMD (g/cm^2^)												
Overall	8	0.03 (−0.02, 0.08)	0.198	R	< 0.001	94.2	8	0.02 (− 0.00, 0.05)	0.086	R	< 0.001	94.1
Caucasian	5	0.00 (−0.04, 0.04)	0.917	R	< 0.001	90.2	5	0.00 (−0.02, 0.02)	0.862	R	< 0.001	91.1
Asian	3	0.11 (−0.16, 0.38)	0.414	R	< 0.001	97.8	3	0.07 (−0.07, 0.20)	0.326	R	< 0.001	97.3
Lumbar Spine Z value												
Overall	3	0.22 (−0.40, 0.83)	0.495	R	< 0.001	88.5	3	0.24 (0.00, 0.47)	0.046	R	0.041	68.6
Caucasian	1	0.13 (0.05, 0.21)	0.001	R	–	–	1	0.18 (0.15, 0.21)	< 0.001	R	–	–
Asian	2	−0.28 (−2.58, 2.02)	0.811	R	0.009	85.2	2	−0.23 (− 1.81, 1.36)	0.780	R	0.062	71.3
Femoral Neck BMD (g/cm^2^)												
Overall	6	0.03 (0.01, 0.05)	0.003	R	0.001	75.5	6	0.01 (−0.00, 0.03)	0.057	R	< 0.001	84.7
Caucasian	5	0.03 (0.01, 0.05)	0.009	R	< 0.001	80.4	5	0.01 (−0.00, 0.03)	0.094	R	< 0.001	87.7
Asian	1	0.03 (−0.01, 0.08)	0.110	R	–	–	1	0.01 (−0.02, 0.04)	0.350	R	–	–
Femoral Neck Z value												
Overall	2	−0.38 (−2.56, 1.80)	0.733	R	< 0.001	99.2	2	0.25 (−0.07, 0.58)	0.130	R	0.001	91.6
Caucasian	1	−1.48 (−1.57, −1.39)	< 0.001	R	–	–	1	0.10 (0.07, 0.13)	< 0.001	R	–	–
Asian	1	0.74 (0.37, 1.11)	< 0.001	R	–	–	1	0.43 (0.24, 0.62)	< 0.001	R	–	–
ERα *Pvu*II	PP vs. pp						Pp vs. pp					
	Test of differences			Model	Test of heterogeneity		Test of differences			Model	Test of heterogeneity	
	N	WMD (95% CI)	*P* value		*P* value	I^2^ (%)	N	WMD (95% CI)	*P* value		*P* value	I^2^ (%)
Lumbar Spine BMD (g/cm^2^)												
Overall	10	0.02 (− 0.01, 0.04)	0.216	R	< 0.001	95.5	10	−0.01 (− 0.02, − 0.00)	0.036	R	< 0.001	84.0
Caucasian	5	0.01 (−0.04, 0.06)	0.793	R	< 0.001	95.5	5	−0.02 (− 0.03, 0.00)	0.106	R	< 0.001	84.9
Asian	5	0.03 (−0.02, 0.08)	0.288	R	< 0.001	96.2	5	−0.00 (− 0.02, 0.02)	0.912	R	< 0.001	86.4
Lumbar Spine Z value												
Overall	3	0.11 (−0.55, 0.78)	0.742	R	< 0.001	98.7	3	0.13 (−0.40, 0.67)	0.623	R	< 0.001	95.9
Caucasian	1	−0.07 (− 0.13, − 0.01)	0.031	R	–	–	1	− 0.16 (− 0.20, − 0.12)	< 0.001	R	–	–
Asian	2	0.24 (−1.72, 2.20)	0.809	R	< 0.001	99.0	2	0.34 (−1.18, 1.85)	0.665	R	< 0.001	97.9
Femoral Neck BMD (g/cm^2^)												
Overall	8	−0.04 (− 0.09, 0.01)	0.135	R	< 0.001	99.3	8	−0.02 (− 0.04, 0.01)	0.132	R	< 0.001	98.2
Caucasian	5	−0.06 (− 0.16, 0.05)	0.295	R	< 0.001	99.6	5	−0.03 (− 0.05, 0.00)	0.054	R	< 0.001	95.2
Asian	3	−0.01 (− 0.02, − 0.01)	< 0.001	R	1.000	0.00	3	−0.00 (− 0.03, 0.02)	0.768	R	0.009	78.7
Femoral Neck Z value												
Overall	2	−0.39 (−1.15, 0.37)	0.315	R	< 0.001	97.0	2	−0.39 (− 0.57, − 0.20)	< 0.001	R	0.024	80.3
Caucasian	1	−0.01 (− 0.08, 0.05)	0.718	R	–	–	1	−0.31 (− 0.35, − 0.27)	< 0.001	R	–	–
Asian	1	−0.79 (−1.05, − 0.53)	< 0.001	R	–	–	1	− 0.50 (− 0.66, − 0.34)	< 0.001	R	–	–

*R* Random effect model

*F* Fixed effect model

#### ERα *Xba*I and femoral neck bone mineral density (BMD g/cm^2^ and BMD Z value)

**Fig. 4:**
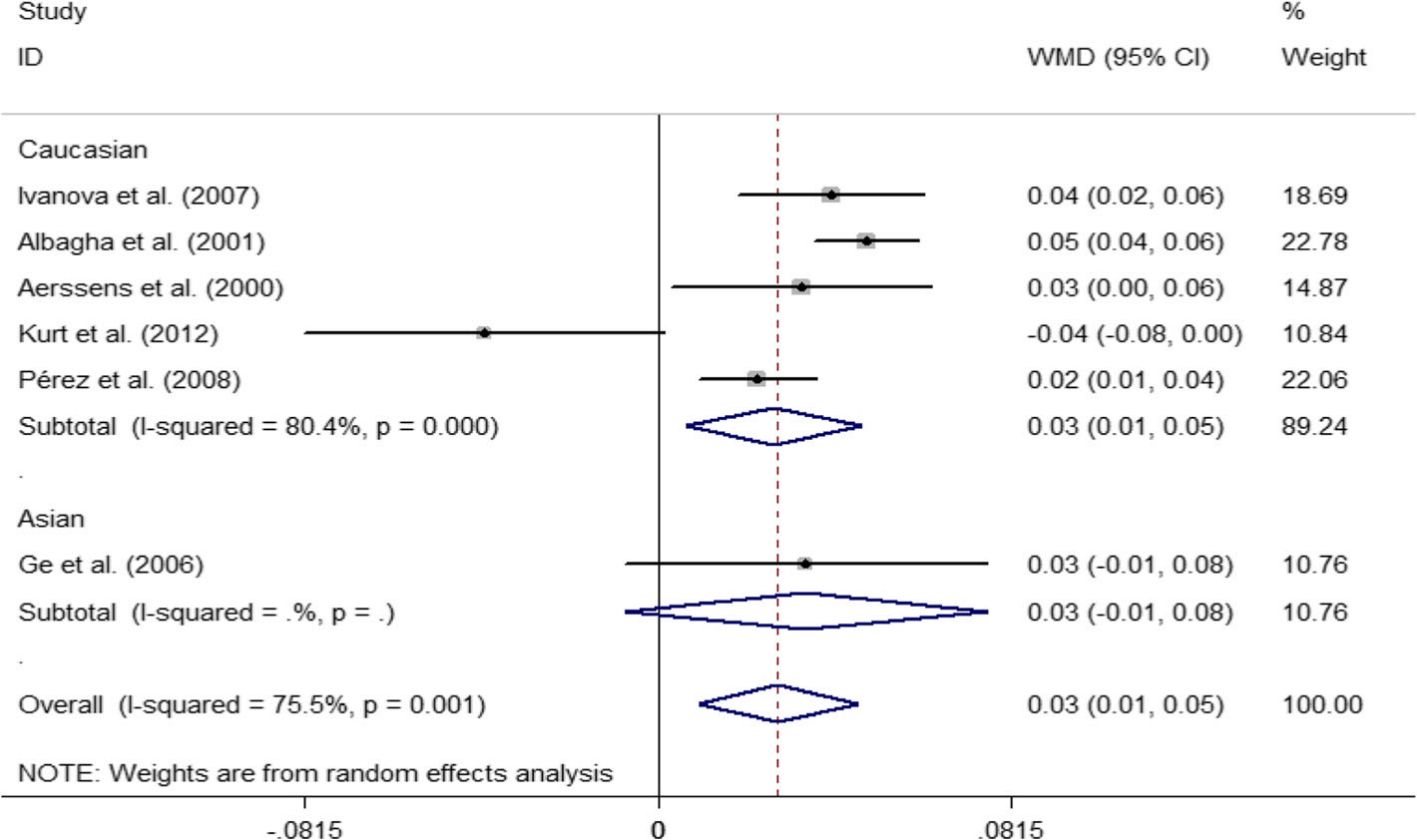
Forest plot showed that XX genotype of ERα *Xba*I was associated with increased femoral neck BMD compared with xx genotype

Our pooled analyses indicated that the ERα *Xba*I XX genotype was significantly associated with increased femoral neck BMD in overall and Caucasian populations. In contrast, ERα *Xba*I XX genotype did not play a key role in femoral neck BMD in Asian populations (Table 5 and Fig. 4). Interestingly, compared with PMOP women with xx genotype, XX genotype was significantly associated with decreased femoral neck Z value in Caucasians, and increased femoral neck Z value in Asians (Table 5). However, no significant association was observed between XX genotype and the femoral neck Z value in overall populations. In addition, Caucasians and Asians carrying the ERα *Xba*I Xx genotype were at risk of a high femoral neck Z value, while no significant association was found in overall populations. We did not observe remarkable relationships between ERα *Xba*I Xx genotype and femoral neck BMD in either overall, Caucasian or Asian populations (all *P* > 0.05). All data are shown in Table 5.

#### ERα *Pvu*II and lumbar spine bone mineral density (BMD g/cm^2^ and BMD Z value)

**Fig. 5:**
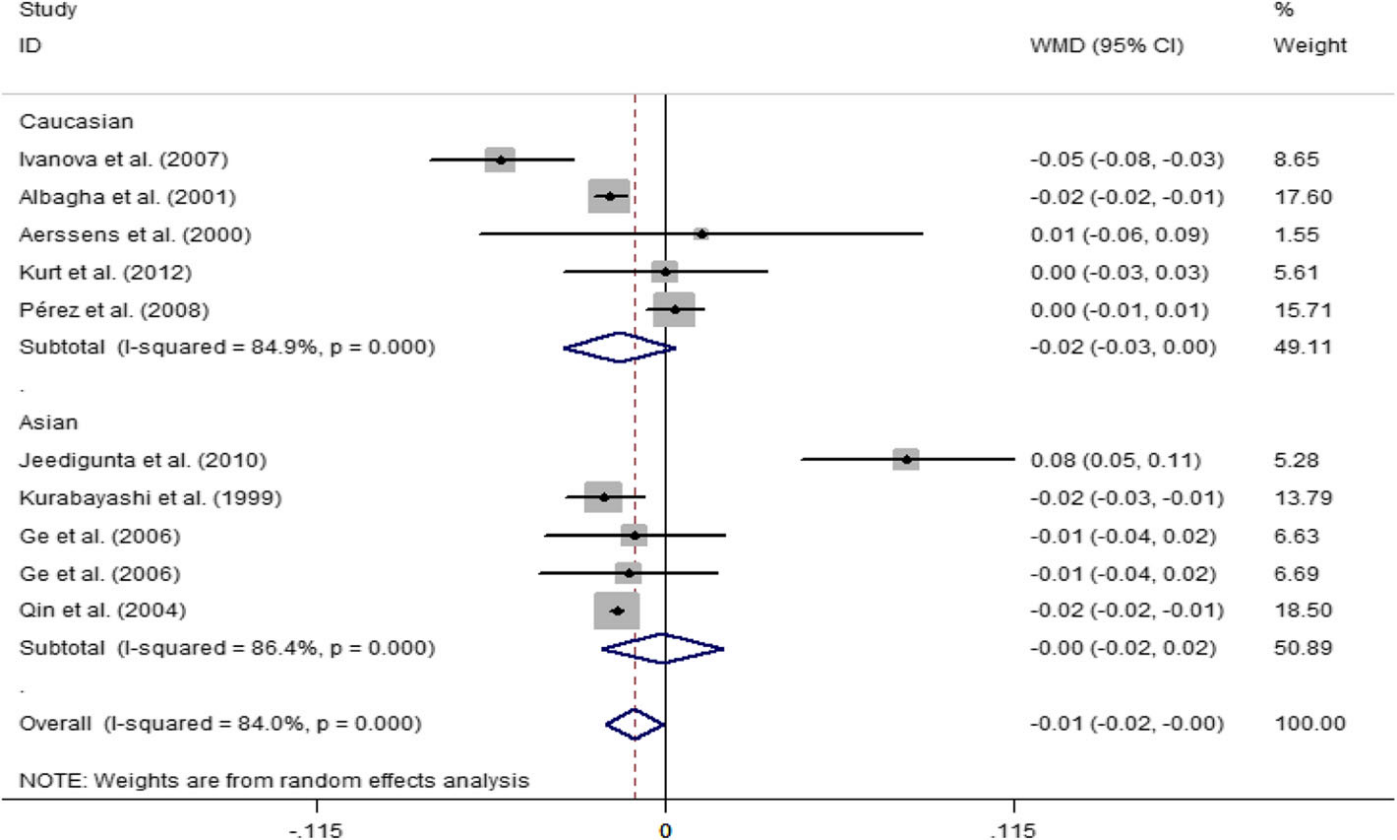
Forest plot showed that Pp genotype of ERα *Pvu*II was associated with increased lumbar spine BMD compared with pp. genotype

With regard to ERα *Pvu*II, the difference in the lumbar spine Z value between the PP and pp. genotypes was − 0.07 (95% CI = − 0.03 to − 0.01, *P* = 0.031) in Caucasian PMOP women; however, no significant difference was observed in overall and Asian populations. For the Pp versus pp. genotype, the difference in lumbar spine BMD was − 0.01 (95% CI = − 0.02 to − 0.00, *P* = 0.036) in overall populations, and the difference in the lumbar spine Z value was − 0.16 (95% CI = − 0.20 to − 0.12, *P* < 0.001) in Caucasian populations; however, we did not find any significant difference in lumbar spine BMD in either Caucasians or Asians, and in the lumbar spine Z value in overall and Asian populations (Table 5 and Fig. 5). In addition, no significant difference in lumbar spine BMD was observed between PP and pp. genotypes (*P* > 0.05) (Table 5).

#### ERα *Pvu*II and femoral neck bone mineral density (BMD g/cm^2^ and BMD Z value)

We further found that the ERα *Pvu*II PP genotype was associated with decreased femoral neck BMD and Z value compared with the pp. genotype in Asians, while no significant difference in femoral neck BMD and Z value was observed in either overall and Caucasian populations (both *P* > 0.05) (Table 5). Furthrmore, PMOP women carrying the Pp genotype were at risk of a low femoral neck Z value, which was found in overall, Caucasian and Asian populations. Our study showed that there was no significant difference in femoral neck BMD between PMOP women with the Pp genotype and those with the pp. genotype (*P* > 0.05). All the data are shown in Table 5.

### Sensitivity analysis and publication bias

**Fig. 6:**
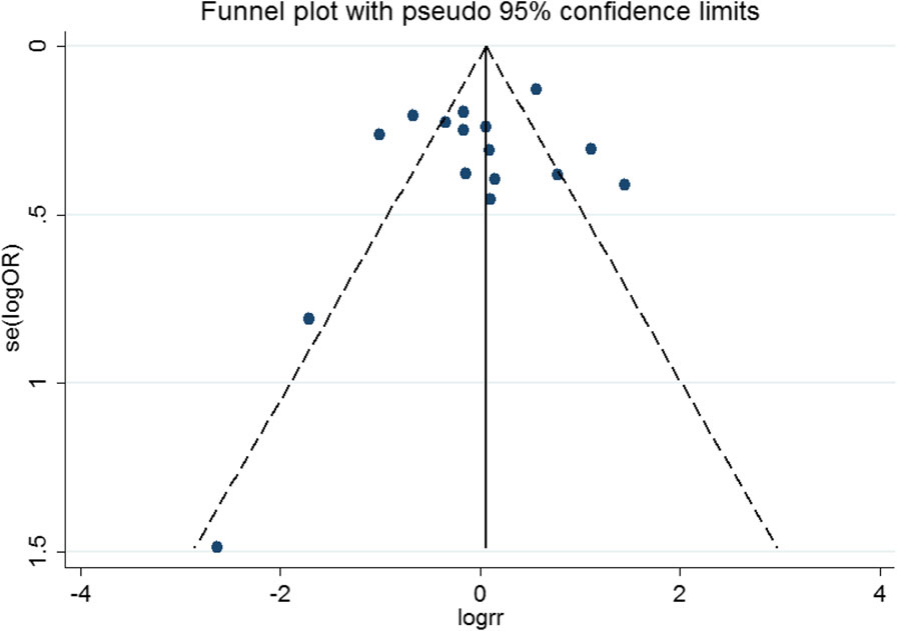
Funnel plot of the ERα *Pvu*II polymorphism and PMOP risk

We performed a leave-one-out analysis to estimate the sensitivity of our study and found that omission of any single study did not affect the overall statistical significance, indicating that the results of our meta-analysis are stable. Therefore, we could conclude that our meta-analysis data are relatively stable and credible. To estimate the publication bias of our meta-analysis, the Begg’s and Egger’s test was performed (Table 4), indicating that there was minimal evidence of publication bias. The shape of funnel plot was symmetrical, which also showed no publication bias in our study (Fig. 6).

## Discussion

### Associations between ERα gene polymorphisms and PMOP risk

ERα *Xba*I and ERα *Pvu*II are the two restriction fragment length polymorphisms of ERα gene located in Intron 1 [14]. Many studies [11–25, 32] have been performed to explore the relationships between ERα *Xba*I, ERα *Pvu*II and PMOP risk; however, these studies have yielded inconsistent data [11–25, 32]. Overall, we did not observe any significant association between ERα *Xba*I and ERα *Pvu*II polymorphisms and PMOP risk in either overall, Caucasian or Asian populations. In our opinion, the inadequate sample size, different ethnicities, various genotyping techniques, the presence of admixture in the population, gene-environment interactions, differences in age and measurement errors of different investigators might be important factors contributing to these controversial results. ERα *Xba*I and ERα *Pvu*II have proven to play key roles in attainment and maintenance of peek bone mass during young adulthood, and it might be difficult to document their effects in a population of postmenopausal women [24]. In addition, *Pvu*II and *Xba*I polymorphisms are located in a non-functional area of the ER gene [20], which might also contribute to our polled results. With regard to ERα G2014A, it is located on the exon region of chromosome 6p25.1, and may contribute via the epigenetic level for the efficiency of translation or receptor protein expression [26]. Our results showed that a significant association between ERα G2014A and PMOP risk was observed only in Caucasian populations but not in overall and Asian populations.

### Associations between ERβ gene polymorphisms and PMOP risk

ERβ has been found to be more abundant than ERα in trabecular bone, and more potent than ERα in mediating estrogen-induced repression of TNF-α expression, which is considered an important contributor to PMOP [30]. ERβ *Alu*I is one of the widely-studied ERβ gene polymorphisms, knowing that it could alter mRNA stability and protein levels, leading to reduced synthesis of ERβ [30]. In our study, ERβ *Alu*I was found to be significantly associated with increased risk of PMOP in Asian populations, while no significant relationship was observed in overall and Caucasian populations. Thus, different genetic backgrounds, environmental effects and/or their internal interactions could explain the diverse results in various ethnicities. ERβ *Rsa*I is another important polymorphism of ERβ. Our subgroup analysis revealed a significant association between ERβ *Rsa*I and PMOP risk in overall populations, which is consistent with the studies of Shoukry et al. [30], and Huang et al. [31].

### Associations between ERα *Xba*I and lumbar spine and femoral neck BMD

Our pooled results showed that there was no significant difference in lumbar spine BMD between PMOP women carrying XX, Xx and xx genotype in either overall, Caucasian or Asian populations. However, WANG et al. [39] reported that the *Xba*I polymorphism was significantly associated with BMD of the lumbar spine, and XX had a protective effect in comparison with carriers of the x alleles, which is consistent with the report of Ioannidis et al. [41]. Both WANG and Ioannidis included all types of osteoporotic patients, not only postmenopausal women, which might be the most important reason for the difference between our results and theirs. As mentioned above, ERα *Xba*I might not play a key role in attainment and maintenance of peek bone mass in postmenopausal women [24], and therefore it could be easily understood why no significant association was observed between ERα *Xba*I and lumbar spine BMD. With regard to femoral neck BMD, our study indicated that the femoral neck BMD in PMOP women with XX genotype was significantly higher than that in women with xx genotype in overall and Caucasian populations, which highlights the theory that ERα gene is involved in the pathogenesis of PMOP. No significant difference of femoral neck BMD was observed between PMOP women with Xx and xx genotype in each subgroup. Although no significant association was observed between lumbar spine BMD and ERα *Xba*I, we found that the lumbar spine Z value in both PMOP women carrying XX and those carrying Xx genotype was significantly higher than that in Caucasians carrying xx genotype. We also observed that XX genotype was associated with a low femoral neck Z value in Caucasians and high femoral neck Z value in Asians. In addition, Caucasians and Asians carrying Xx genotype were at risk of a high femoral neck Z value. However, why ERα *Xba*I plays a contradictory role in BMD and Z value at the lumar spine and femoral neck, and the mechanisms by which it is associated with BMD and Z value remains unclear and needs further investigation.

### Associations between ERα *Pvu*II and lumbar spine and femoral neck BMD

Although the molecular mechanism underlying the effect of ERα *Pvu*II on bone mass is poorly understood, it is believed that ERα *Pvu*II might play a key role in BMD as it is in linkage disequilibrium with the TA polymorphism in the ER promoter that is associated with altered gene transcription [20]. Our pooled analysis indicated that PMOP women with the Pp genotype had lower lumbar spine BMD than those with the pp. genotype. We also found that there was no significant difference in lumbar spine BMD between women with the PP genotype and those with the pp. genotype, which is consistent with the meta-analysis of Wang et al. [40]. Furthermore, we observed that the PP genotype was associated with decreased femoral neck BMD in Asians, while Pp might not play a key role in femoral neck BMD in all subgroups. Interestingly, WANG et al. [39] reported that PP play a role in protecting the lumbar spine but on the other hand it might be a risk factor for the femoral neck fracture. Wang CL [40] and WANG KJ [39] conducted their meta-analyses on osteoporotic women during menopause while our study included osteoporotic women post menopause, which might be the most important reason for the difference between our study and theirs. In addition, both PP and Pp genotypes were significantly associated with low lumbar spine Z value in Caucasians, but not in overall and Asian populations, probably because of the different genetic backgrounds in various ethnicities and interactions between genetic and non-genetic factors. PMOP women with the PP and Pp genotypes had lower femoral neck Z value than those with the pp. genotype in overall, Caucasian and Asian populations.

### Limitations

Although we performed a comprehensive analysis of the association between ERα, ERβ gene polymorphisms and PMOP risk and BMD in postmenopausal women, there are some limitations that should be addressed. First, high heterogeneity was observed in some of our pooled results, which might have negative impact on our conclusions. Second, PMOP is a disease whose etiology might be involved in several confounding factors, and other confounding factors such as age, years since menopause and estrogen therapy might interact with each other and play a key role in the etiology and progression of PMOP. However, no data available could be used in all recruited studies to detect the interactions between these confounding factors in PMOP patients. We should take all these confounding factors into consideration in our study rather than studying them separately, which is also a limitation of our meta-analysis. Third, we failed to perform a pooled analysis to detect whether ERα G2014A, ERβ *Alu*I and ERβ *Rsa*I were correlated with BMD in postmenopausal women as no sufficient data could be collected and analyzed. Therefore, larger-scale and better-designed studies are necessary to determine the association between ERα/β gene polymorphisms and PMOP risk and BMD in postmenopausal women.

## Conclusion

ERα/β gene polymorphisms were significantly associated with PMOP risk and BMD in postmenopausal women, but each ERα/β gene polymorphism may have a distinct effect on PMOP risk and BMD in Asian and Caucasian populations.

## Abbreviations

BMD: Bone mineral density
CI: Confidence interval
ER: Estrogen receptor
Lactase: LCT
OR: Odds ratios
PMOP: Postmenopausal Osteoporosis
PTH: Parathyroid Hormone
TGF-β: Transforming growth factor-β
WMD: Weight mean difference
